# Factors associated with the intention of pregnant women to give birth with epidural analgesia: a cross-sectional study

**DOI:** 10.1186/s12884-023-05887-w

**Published:** 2023-08-22

**Authors:** Luka Van Leugenhaege, Julie Degraeve, Yves Jacquemyn, Eveline Mestdagh, Yvonne J. Kuipers

**Affiliations:** 1 Department of Health and Life Science, School of Midwifery, AP University of Applied Sciences and Arts Antwerp, Noorderplaats 2, 2000 Antwerp, Belgium; 2 Faculty of Medicine & Health Sciences, Department of Nursing and Midwifery, University of Antwerp, Universiteitsplein 1, 2610 Wilrijk, Belgium; 3 Department of Obstetrics and Gynecology, University Hospital Antwerp UZA, Drie Eikenstraat 655, 2650 Edegem, Belgium; 4 University of Antwerp, ASTARC and Global Health Institute GHI, Universiteitsplein 1, 2610 Wilrijk, Belgium; 5 School of Health and Social Care, Edinburgh Napier University, 9 Sighthill Court, Edinburgh, EH11 4BN Scotland

**Keywords:** Epidural analgesia, Anxiety, Obstetric Labour, Personality inventory, Pregnancy, Midwifery

## Abstract

**Background:**

In Belgium most women receive epidural analgesia during labour. Although, it offers satisfactory pain relief during labour, the risk on a series of adverse advents has been reported. The objective of this study was to determine factors associated with the intention of pregnant women, anticipating a vaginal birth, of requesting epidural analgesia during labour.

**Methods:**

A cross-sectional study, using an online self-report questionnaire was performed, including socio-demographic and personal details. Associated factors were examined with the HEXACO-60 questionnaire, the Mental Health Inventory-5, the Tilburg Pregnancy Distress Scale and the Labour Pain Relief Attitude Questionnaire for pregnant women. The level of intention to request epidural analgesia was based on two questions: Do you intend to ask for epidural analgesia (1) at the start of your labour; (2) at some point during labour? Data were collected predominantly during the second and third trimester of pregnancy. Descriptive analysis and a multiple linear regression analysis were performed.

**Results:**

949 nulliparous (45.9%) and multiparous (54.1%) pregnant women, living in Flanders (Dutch-speaking part of Belgium) anticipating a vaginal birth completed the questionnaires. Birth-related anxiety (ß 0.096, p < 0.001), the attitude that because of the impact of pregnancy on the body, asking for pain relief is normal (ß 0.397, p < 0.001) and feeling more self-confident during labour when having pain relief (ß 0.034, p < 0.001) show a significant positive relationship with the intention for intrapartum epidural analgesia. The length of the gestational period (ß − 0.056, p 0.015), having a midwife as the primary care giver during pregnancy (ß − 0.048, p 0.044), and considering the partner in decision-making about pain relief (ß − 0.112, p < 0.001) show a significant negative relationship with the intention level of epidural analgesia. The explained variability by the multiple regression model is 54%.

**Conclusions:**

A discussion during pregnancy about the underlying reason for epidural analgesia allows maternity care providers and partners to support women with pain management that is in line with women’s preferences. Because women’s intentions vary during the gestational period, pain relief should be an issue of conversation throughout pregnancy.

## Background

An international study on childbirth interventions in high-income countries reveals that use of epidural analgesia (EA) during labour varies across countries, for nulliparous women from 19.4 to 83.4% and 10.0–64.4% in multiparous women [1]. In Flanders, the Dutch-speaking part of Belgium, 66.7% of all childbearing women with a vaginal birth has EA during labour, 77.1% among nulliparous women and 58.5% among multiparous women. Within Europe, intrapartum EA in Belgium is the highest and similar to the uptake in the United States [1–5].

Despite the possible adverse effects of EA (e.g. maternal hypotension, prolonged labour, increased risk of augmentation of labour) [2–4], the Belgian national guidelines on intrapartum care recommend EA as the most effective method of pain relief during birth and it is accepted as the norm among Flemish women [6, 7]. However, a discrepancy has been reported between Flemish women’s intention to give birth with EA and the actual uptake of intrapartum EA [8]. When women do not receive intrapartum EA in accordance with their initial intention, it is likely to contribute to a negative birth experience and to increase the risk of developing postpartum depression [9, 10], whilst when the intention and actual uptake match, women are more likely to have positive birth experiences [11].

Several factors are known to be associated with wanting intrapartum EA, such as prior epidural, partner preferences, type of care professional, accepting EA as the norm and self-confidence [2, 11, 12]. Additionally, women’s choice for intrapartum pharmacological pain relief is associated with birth-related anxiety and with a history of psychological problems [12–14]. Also, women with overall low levels of extraversion, agreeableness, emotional stability, conscientiousness, and openness to experiences are more likely to use pharmacological pain relief during labour [15, 16]. Certain personality traits (e.g. agreeableness, extraversion) are associated with birth-related anxiety, childbirth experience and intrapartum interventions such as pharmacological pain relief.

Although various studies have identified individual factors that affect the intention for EA during birth [12, 13, 16, 17], no research has been performed to explore to what extent factors such as mental health, birth-related anxiety, personality traits and attitudes towards pain relief contribute to Flemish women’s intentions about EA. This knowledge would allow healthcare professionals to support women in sustaining their intention to choose or not to choose EA. Therefore, the aim of this study was to determine the factors associated with the intention of Flemish pregnant women who are anticipating a vaginal birth, to request EA during labour.

## Methods

### Design and procedure

This study was conducted among pregnant women in Flanders in a descriptive and cross-sectional manner. Eligible women were 18 years or older, during any trimester of pregnancy and any parity. Data were collected between January and May 2020, using an online self-report questionnaire (Lime Survey©) in the Dutch language. Non-probability sampling techniques were applied: Convenience sampling included informing health care professionals providing prenatal care to Flemish women (i.e. midwives, obstetricians, doula’s, physiotherapists) about the study and asking them to distribute flyers and posters to potential participants – acting as gatekeepers for recruitment. Participants were also recruited via social media platforms, allowing snowballing. A link or Quick Response (QR)-code anonymously redirected participants to the questionnaire. The non-probability sampling technique was applied to reach as many pregnant women in Flanders as possible, to achieve a representative distribution of women in maternity settings, as the EA rate varies between Flemish sites [18].

### Measures

Intention was measured with two items: (i) Do you intend to ask for EA at the start of your labour; (ii) Do you intend to ask for EA at some point during labour? Participants could answer the questions with: ‘yes,’ ‘undecided’, or ‘no’. The Dutch versions of the Tilburg Pregnancy Distress Scale (TPDS), the Labour Pain Relief Attitude Questionnaire for pregnant women (LPRAQ-p), and the validated Dutch versions of the HEXACO-60 and Mental Health Inventory (MHI-5) were included. These four existing self-report measures were completed to explore the impact of personality traits (HEXACO-60), general and pregnancy/birth specific psychological issues (MHI-5 and TPDS) and attitudes towards intrapartum pain relief (LPRAQ-p).

#### HEXACO-60

The HEXACO-60 questionnaire assesses six personality traits: Honesty-Humility, also known as integrity (H), Emotionality (E), Extraversion (X), Agreeableness (A), Conscientiousness (C), and Openness to Experience (O) via agreement on a five-point scale (1 = strongly disagree, 5 = strongly agree) to ten self-descriptive statements per trait. The HEXACO domains have shown good internal consistency (Cronbach’s Alpha α 0.73-0.79) [18].

#### MHI-5

The MHI-5 is the mental health subscale of the 36-item Short-Form Health Survey Questionnaire (SF-36) [19], a brief, valid and reliable tool for detecting psychological wellbeing in the general population [20], showing good internal consistency (α.85) in a Dutch-speaking population [21]. The MHI-5 consists of five questions, asking how participants felt the last four weeks. Each item has six response options ranging from ‘all the time’ (*0*) to ‘at no time’ (*5*). After computing the MHI-5 values (multiplying each score by four) the total score varies from 0 to 100, with higher scores indicating good mental health [22].

#### TPDS

The TPDS assesses pregnancy distress and consists of 16 items and two subscales as it explores the negative affect related to the woman’s pregnancy and birth (TPDS-NA-11 items) and the woman’s perception of partner involvement (TPDS-PI-5 items). The TPDS-NA includes five specific birth-related items referred to as TPDS-C (confinement). The TPDS uses a 4-point rating scale generating a total score ranging from 0 to 48. A total TPDS score above 17 indicates an increased negative affect towards pregnancy and birth. A total TPDS-NA score above 12 indicates birth-related anxiety and fear [23]. The TPDS was originally developed and validated for a Dutch speaking population. It showed good psychometric properties and good internal consistency (α.78, α.80) for the 16-item scale and acceptable internal consistency (α.71) for the 11-items TPDS-NA scale [23–25].

#### LPRAQ-p

The Labour Pain Relief Attitude Questionnaire for pregnant women consists of 6 items formulated as statements, using a five-point score ranging from ‘totally disagree’ (*1*) to ‘totally agree’ (*5*). The questionnaire was originally developed for a Dutch speaking pregnant population and was validated by 850 Dutch childbearing women showing acceptable internal consistency (α.76) and a good model fit [26, 27].

### Analysis

An a priori sample size calculation, based on the number of women who gave birth in 2019 in Flanders, was conducted with statistical significance set at *p* 0.05 (95% CI) [28]. This showed a required minimum of 382 participants. A power analysis was performed using G-Power 3.1© to justify the number of covariates, showing an estimated moderate effect size (0.13) and power of 80% [29]. Normality of distribution was checked using visual interpretation of histograms and QQ-plots. We calculated descriptive statistics for the personal characteristics. When > 10% of the values per case were missing, the values were not imputed. Cronbach’s alpha was calculated to measure internal consistency of the HEXACO categories, MHI-5 and the TPDS. The results were considered as low at α < 0.7, acceptable at 0.7 ≤ α < 0.8, good at α 0.8 ≤ α < 0.9 and excellent at α ≥ 0.9 [30]. Sum scores were calculated for the six HEXACO domains, MHI-5, TPDS 16 items, TPDS-NA and for TPDS-C.

The answers for intention of EA during birth were recoded. When participants answered ‘no’ on both questions if they intended to have EA during labour, either from the start or at some point later during birth, this was recoded in *0*. ‘No’ from the start of birth and ‘undecided’ later during birth, was recoded in *1*. When participants answered ‘undecided’ on both questions this was recoded in *2*. ‘No’ for intending to have EA from the start of birth but ‘yes’ for intending epidural analgesia at some point later during the birth, was recoded in *3*. ‘Undecided’ for EA at the start of birth but ‘yes’ for at some point later during birth was recoded in *4*. When answering ‘yes’ on both questions this was recoded in *5*. This way, a continuous variable (intention EA) was constructed to be used as the dependent variable in the multiple linear regression analysis (MLRA). The lower the score, the lower the intention to use EA during labour. MLRA was used to examine the relationship between the dependent variable (intention EA) and the multiple independent variables (ethnic background, age, partnership, level of education, working status, gestation, gravidity, parity, history of miscarriage, progress pregnancy, primary caregiver, personal history of psychological problems, six HEXACO domains, MHI-5, TPDS-NA, TPDS-PI, the TPDS-C, LPRAQ-p). The independent variables were selected based on the literature [2, 12–14, 16, 17, 31, 32]. The correlation matrix was checked for multicollinearity of the independent variables. To enter the independent variables (n = 29) in the MLRA, we needed a minimum sample of 870 participants [30]. The analyses were performed using the Statistical Package for the Social Sciences© (SPSS) version 26.

## Results

### Participants

**Fig. 1:**
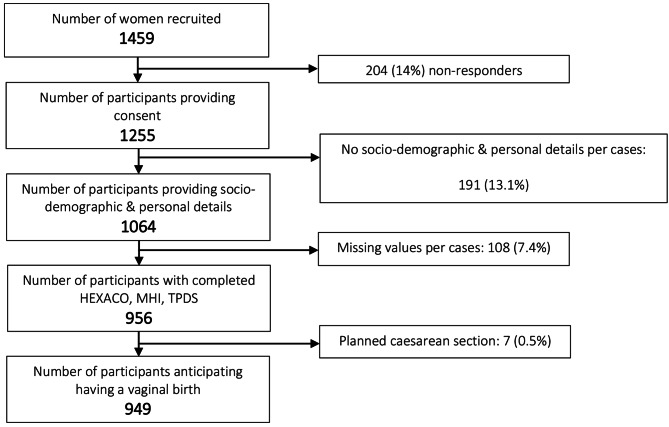
Flowchart

A total of 949 women were included in the study (see Fig. 1). Most of the participants had a Belgian background, were cohabiting, had high levels of education and a job. Participants were predominantly in the second and third trimester of pregnancy. There were more multiparous women (54.1%) in the sample than nulliparous women (45.9%), with an overall healthy self-reported progress of pregnancy. Most of the participants received obstetrician-led antenatal care (84.5%). Around a third of the sample reported reduced psychological wellbeing and an increased negative affect towards pregnancy and birth. About one in every four multiparous women reported a history of negative or traumatic birth experience(s). Most multiparous women (65.9%) had used intrapartum EA before. The characteristics and personal details of the respondents are presented in Table 1.

**Table 1:** Socio-demographic and personal details participants (n = 949)

	N (%)	Mean (SD±) range
**Age** in years		29.4 (±3.7) 20–44
**Ethnic background**		
Born in Belgium	907 (95.6)	
Not born in Belgium	42 (4.4)	
**Partnership**		
Cohabiting	925 (97.5)	
Not cohabiting	24 (2.5)	
**Highest level of education**		
Elementary school	11 (1.2)	
(Pre-vocational) secondary education	278 (29.3)	
Higher education, including bachelor (equivalent)	437 (46)	
University	223 (23.5)	
**Job**		
Paid job	862 (90.8)	
Full-time	665 (77.1)	
Part-time	197 (22.9)	
No (paid) job	65 (6.9)	
Student	22 (2.3)	
**Planned pregnancy**		
Yes	849 (88.5)	
No	100 (11.5)	
**Gestation** in weeks		27.6 (±8) 12–42
**Self-reported progress of pregnancy**		
Good/healthy	846 (88.1)	
Not so good/Complications	103 (10.9)	
**Trimester of pregnancy**		
First trimester (0–12 weeks)	14 (1.5)	
Second trimester (13–26 weeks)	411 (43.3)	
Third trimester (27–42 weeks)	524 (55.2)	
**Parity**		
Nulliparous	436 (45.9)	
Multiparous	513 (54.1)	
**Past birth experience and epidural analgesia**		
Parous women (n = 513) with epidural analgesia during previous birth	338 (65.9)	
Parous women (n = 513) with a self-reported negative or traumatic birth experience	131 (25.5)	
**History of pregnancy loss**		
Yes	54(5.6)	
No	895 (94.4)	
**Primary caregiver antenatal period**		
Obstetrician	802 (84.5)	
Independent community midwife	119 (12.5)	
Shared antenatal care obstetrician & midwife	14 (1.5)	
General practitioner	14 (1.5)	
**Personal history of psychological problems (diagnosed)**		
Yes	119 (12.5)	
No	830 (87.5)	
**HEXACO-60**		
Integrity (α.77)		3.6 (±0.48) 1.9–4.9
Emotionality (α.75)		3.36 (±0.55) 1.6–4.8
Extraversion (α.79)		3.40 (±0.54) 1.3–4.8
Agreeableness (α.70)		3.05 (±0.52) 1-4.4
Conscientiousness (α.70)		3.68 (±0.52) 1.4-5
Openness to experience (α.77)		2.91 (±0.62) 1-4.6
**MHI-5** (α.83)		
Total score		67.68 (±13.52) 16–100
Below cut-off (≤ 60)	279 (29.4)	
**TPDS**		
Total score TPDS 16-items (α.81)		15.63 (±7.37) 0–43
Above cut-off (> 17)	333 (35.1)	
Total score TPDS-NA 11 items (α.78)		10.94 (±6.23) 0–33
Above cut-off (> 12)	316 (33.3)	
Total score TPDS-PI 5 items (α.83)		4.69 (±3.1) 0–15
Total score TPDS-C 5 items (α.86)		4.53 (±3.6) 0–15

Most women indicated not wanting an EA at onset of labour (64.7%). The answers about wanting an EA at some point during the birth showed that an equal proportion of women want or do not want EA and a slightly higher percentage remains undecided (Table 2).

**Table 2:** Intention and attitude epidural analgesia during birth (n = 949)

	N (%)	Mean (SD±) range
**Level of intention for use of epidural**		1.81 (±1.8) 0–5
**Do you intend to ask for EA at the start of your labour?**		
No	614 (64.7)	
Yes	127 (13.4)	
Undecided	208 (21.9)	
**Do you intend to ask for EA at some point during labour?**		
No	294 (31)	
Yes	302 (31.8)	
Undecided	353 (37.2)	
LPRAQ-p^*^ (α.72)		
1. Because my pregnancy has already had a big impact on my body, I think it is normal to ask for pain relief.		2.68 (±1.2) 1–5
2. I also ask for pain relief because of my partner.		1.52 (±0.8) 1–5
3. I am convinced that if I get pain relief, I will feel much more self-confident during labour.		2.44 (±1.3) 1–5
4. Pain relief will help me perform much better during labour.		2.49 (±1.2) 1–5
5. My partner plays an important role in the decision to ask for pain relief during labour.		1.9 (±1.1) 1–5
6. My (social) environment (friends, relatives) plays an important role in the decision to ask for pain relief during labour.		**

^*^ 1 = totally disagree, 5 = totally agree; ** >10% missing values

### Associated factors

The scales HEXACO-60 subscales, MHI-5, TPDS, TPDS-NA, TPDS-PI, TPDS-C, and LPRAQ-p showed acceptable to good internal consistency. The following independent variables showed multicollinearity: TPDS-NA and TPDS-C (*r*.86), gravidity and parity (*r*.80), item 3 and 4 of the LPRAQ-p (*r*.80) and midwife and obstetrician as primary care giver during pregnancy (*r*.88). The TPDS-NA score, gravidity, item 4 (I will perform better during labour when I have pain relief) and obstetrician-led care were removed from the analysis. Two cases showed residual > 3. With a large sample size, Cook’s distance < 1 and Mahala Nobis’ distance < 15, and re-running the analysis without the cases showing no differences in results. The cases were kept in MLRA [30]. MLRA shows that birth-related anxiety (ß 0.096, p < 0.001), the attitude that because of the impact of pregnancy on the body, it is normal to ask for pain relief (ß 0.397, p < 0.001), being convinced to feel more self-confident during labour when having pain relief (ß 0.034, p < 0.001) have a significant positive relationship with the intention level of EA. The length of the gestational period (ß − 0.056, p 0.015), having a midwife as the primary care giver during pregnancy (ß − 0.048, p 0.044) and the partner playing a significant role in deciding to request pain relief (ß − 0.112, p < 0.001) have a significant negative relationship with the intention for EA. The amount of explained variability by the regression model is 54% (Table 3).

**Table 3:** Multiple linear regression analysis of predictors of intention for epidural analgesia during labour (n = 949)

						95% Confidence Interval (CI) for B		
	B	SE B	ß	t	Sig (p)	Lower bound	Upper bound	
(Constant)				-1.241	0.215	-2.553	0.575	
Born in Belgium	0.107	0.196	0.013	0.546	0.585	− 0.277	0.491	
Age (in years)	0.015	0.012	0.031	1.232	0.218	− 0.009	0.038	
Level of participation in (un)paid job or study	− 0.275	0.174	− 0.042	-1.582	0.114	− 0.617	0.066	
Cohabiting (vs. not cohabiting)	− 0.178	0.118	− 0.050	-1.505	0.133	− 0.411	0.054	
Level of education	− 0.009	0.024	− 0.010	− 0.385	0.7	− 0.056	0.037	
Planned pregnancy (planned vs. unplanned)	− 0.164	0.137	− 0.029	-1.203	0.229	− 0.433	0.104	
Length gestational period (in weeks)	− 0.012	0.005	− 0.056	-2.425	0.015^**^	− 0.022	− 0.002	
Parity (number of births)	− 0.097	0.062	− 0.039	-1.551	0.121	− 0.219	0.026	
History of miscarriage (no vs. yes)	− 0.327	0.242	− 0.031	-1.354	0.176	− 0.802	0.147	
Progress pregnancy (good vs. not so good)	− 0.038	0.133	− 0.007	− 0.285	0.775	− 0.299	0.223	
History of psychological problems (no vs. yes)	− 0.184	0.124	− 0.035	-1.479	0.140	− 0.428	0.060	
Integrity	0.033	0.118	0.009	0.282	0.778	− 0.198	0.264	
Emotionality	− 0.034	0.081	− 0.011	− 0.421	0.674	− 0.193	0.125	
Extraversion	0.012	0.082	0.004	0.148	0.883	− 0.149	0.174	
Agreeableness	− 0.008	0.081	− 0.002	− 0.098	0.922	− 0.167	0.151	
Conscientiousness	0.126	0.080	0.037	1.580	0.114	− 0.030	0.283	
Openness to experiences	− 0.121	0.069	− 0.043	-1.754	0.080	− 0.256	0.014	
MHI-5 (mental health)	0.003	0.004	0.027	0.938	0.349	− 0.004	0.011	
TPDS-C (birth-related anxiety)	0.047	0.012	0.096	3.801	< 0.001^*^	0.023	0.071	
TPDS-PI (level of partner involvement)	0.019	0.014	0.033	1.299	0.194	− 0.010	0.047	
Midwife as primary care giver	− 0.256	0.127	− 0.048	-2.018	0.044^**^	− 0.506	− 0.007	
Because my pregnancy has already had a big impact on my body, I think it is normal to ask for pain relief.	0.578	0.043	0.397	13.441	< 0.001^*^	0.494	0.653	
My request for pain relief is also because of my partner.	0.078	0.059	0.034	1.329	0.184	− 0.037	0.193	
I am convinced that if I get pain relief, I will feel much more self-confident during labour.	0.528	0.046	0.341	11.502	< 0.001^*^	0.438	0.618	
My partner plays an important role in the decision to ask for pain relief during labour.	− 0.184	0.041	− 0.112	-4.515	< 0.001^*^	− 0.264	− 0.104	

*(Constant) is level of intention of pregnant women to giving birth with EA;* ^*^ *p < 0.001;* ^**^ *p < 0.05; R* ^*2*^ *0.539*

## Discussion

This study indicates that birth-related anxiety, partner involvement, midwife-led care, length of gestational period and women’s attitude towards labour pain relief affects the level intention for EA during childbirth among Flemish pregnant women. The regression model in our study explains a substantial portion of the determinants of childbearing women’s level of intention to request epidural analgesia during childbirth, offering potential for supporting women in their preferences for intrapartum pain management.

As our model shows that having a midwife as primary caregiver correlates with a lower level of intention for EA, it is relevant to consider the role of the care giver [6, 14]. The health care professional’s attitude towards EA has an impact on women requesting EA during labour, as women value the opinion of their care giver and tend to adopt the care professional’s preference [8]. Our findings show that a relationship between primary care provider and preference for EA exists, suggesting different philosophies of care or opinions are driven by professional background [33]. Midwives aspire to promote and support the natural progress of labour and birth [34], while obstetricians are trained to perform medical interventions. This makes it imperative for obstetricians and midwives to be aware of their own preferences and opinions and to be reflective about how this influences their communication with women during pregnancy. Based on our findings, it can be recommended for the Belgian midwife to take a more prominent position in current antenatal care [6], as this might contribute to support women to pursue their antenatal preferences for intrapartum pain management. This recommendation is transferable to maternity care systems similar to that of Belgium, characterized by a high prevalence of intrapartum interventions such as EA [3–5] and high rates of obstetrician-led care.

According to our findings, not only professionals’ but also the woman’s partner’s opinions matter to childbearing women. Partners’ presence during labour and birth actually reduces the use of pain medication, due to their emotional and practical support leading up to birth [2], underlining the need for antenatal care professionals to support partner-involvement with regard to intrapartum pain management from the onset of pregnancy to labour and birth.

Our findings suggest that attitude, self-confidence, and fear seem essential to understanding the intention to use EA during labour. Perceiving that pregnancy has a an impact on the body and therefore regarding EA during labour as ‘normal’ might refer to the normalization of EA as an expected and safe intervention, but also to fear of pain [10, 35]. When women are fearful of, for example, being overwhelmed by pain during childbirth, it suggests less confidence in own ability to cope with labour pain [35].

Our results show a discrepancy between women’s intrapartum EA intentions and actual use [4]. Women in the study report different levels of EA intention at different stages of pregnancy, suggesting that intentions for EA are lower when the woman is more advanced in her pregnancy. This implies that women become more positive about labour pain and pain acceptance as pregnancy progresses. Information and discussion about women’s intentions of pain management are therefore key and should be addressed throughout pregnancy.

Our study had several strengths such as a large sample size, exceeding the a priori calculated minimum of participants required to make reliable inferences. All scales showed acceptable to good internal consistency. To our knowledge, this is the first study on the influence of personality traits, mental health, birth related anxiety and partner involvement on the level of intention to use EA during labour in a Flemish population. Also, our study is the first study, that we are aware of, to reveal that level of intention for EA can vary depending on gestational length.

Some aspects of the study are subject to discussion. Although our data such as age and parity of the women in our study are congruent with the Flemish perinatal data [28], the findings of our study are only generalizable to women with similar characteristics. Flemish statistics reveal a higher proportion of mothers with a non-Belgian nationality, without a (officially) cohabiting partner and without employment at time of birth [36]. Moreover, the official national database is missing data on mothers level of education (> 20% data missing), making it impossible to compare with our data [36] Albeit our regression model explains a substantial proportion of factors associated with intention for EA, our study design is cross-sectional and therefore the lowest level of the aetiology hierarchy [37].This study does not provide information about causality between actual uptake of EA and the underlying factors reported in this study. Further research including longitudinal cohort studies comparing the level of intention to the received EA during childbirth and how progression of labour, interventions, organization of antenatal care and women’s coping strategies affect the difference between prenatal intention and actual use of EA during labour is needed. Additionally, qualitative research to determine women’s needs in facilitating and fully supporting their pain management intentions during labour would be of merit. Although Belgian guidelines recommend to inform women about pain management during the third trimester [6], we do not know if, what, when, how and by whom women in this study received information on intrapartum pain management. This could have created bias as it is difficult to know how this affected women’s levels of intention.

## Conclusions

Overall, Flemish pregnant women’s intentions for intrapartum pain relief show an incongruence with the actual uptake of EA during labour in Flanders. Midwives might be able to play a vital role in informing and supporting women and their partners on intrapartum pain management throughout pregnancy. Antenatal care should entail discussing women’s intention for intrapartum pain management and how this is affected on an individual level by anxiety towards labour and birth, beliefs and attitudes towards pain and EA. This to strengthen women in their personal preferences towards EA use during labour. Furthermore, partner-involvement should be regarded as a valuable aspect of supporting women’s intentions for intrapartum pain management. A more in-depth discussion during pregnancy about the reasons behind women’s intention for EA will allow maternity health care providers and partners to support a woman in her intention in how to manage pain during labour to potentially avoid incongruence between intentions and expectations. Because women’s intentions vary during the gestational period, indicating perceptions change over time, pain relief should be an issue of conversation throughout pregnancy.

## Data Availability

The datasets used and/or analysed during the current study are available from the corresponding author on reasonable request.

## List of abbreviations

EA: Epidural Analgesia
LPRAQ-p: Labour Pain Relief Attitude Questionnaire for pregnant women
MHI-5: Mental Health Inventory
MLRA: Multiple linear regression analysis
QR: Quick Response
SPSS: Statistical Package for the Social Sciences
TPDS: Tilburg Pregnancy Distress Scale
α: Cronbach’s Alpha
